# Decoupling of inorganic and organic carbon during slab mantle devolatilisation

**DOI:** 10.1038/s41467-022-27970-0

**Published:** 2022-01-14

**Authors:** P. Bouilhol, B. Debret, E. C. Inglis, M. Warembourg, T. Grocolas, T. Rigaudier, J. Villeneuve, K. W. Burton

**Affiliations:** 1 Université de Lorraine, CNRS, CRPG, 54000 Nancy, France; 2 Université de Paris, Institut de physique du globe de Paris, CNRS UMR 7154, 1 rue Jussieu, 75005 Paris, France; 3 Department of Earth Sciences, Arthur Holmes Building, Durham University, South Road, Durham, DH1 3LE UK

**Keywords:** Geochemistry, Petrology

## Abstract

Serpentinites are an important sink for both inorganic and organic carbon, and their behavior during subduction is thought to play a fundamental role in the global cycling of carbon. Here we show that fluid-derived veins are preserved within the Zermatt-Saas ultra-high pressure serpentinites providing key evidence for carbonate mobility during serpentinite devolatilisation. We show through the O, C, and Sr isotope analyses of vein minerals and the host serpentinites that about 90% of the meta-serpentinite inorganic carbon is remobilized during slab devolatilisation. In contrast, graphite-like carbonaceous compounds remain trapped within the host rock as inclusions within metamorphic olivine while the bulk elemental and isotope composition of organic carbon remains relatively unchanged during the subduction process. This shows a decoupling behavior of carbon during serpentinite dehydration in subduction zones. This process will therefore facilitate the transfer of inorganic carbon to the mantle wedge and the preferential slab sequestration of organic carbon en route to the deep mantle.

## Abstract (web summary)

Hydrated mantle rocks store a significant amount of organic and inorganic carbon, impacting the geological cycle. During subduction the carbonate return to the upper plate while organic carbon remains trapped to be recycled in the deep earth.

## Introduction

During subduction, slab devolatilization supplies fluids to the mantle wedge and upper plate, whereas the residual slab is subducted to greater depth, playing a key role in Earth’s geochemical cycle. Much attention has been given to the recycling of H_2_O, with an emphasis on the role of the serpentinized slab mantle which dominates the water budget of subduction systems^1^. In comparison, the carbon budget of subduction systems remains poorly understood, with little consensus on flux estimates^2,3^. Most of the carbon subducted in sediments and the oceanic crust is scavenged by slab dehydration reaction to the overlying mantle wedge during the low-pressure stage of slab devolatilization in the fore-arc region^4–7^. These fluids contribute to the hydration and carbonation of the fore-arc mantle e.g.^8,9^, while the residual slab contributes to the deep earth cycle^10^. Serpentinites have the capacity to hold more than 1 wt% of C, and therefore, represent a substantial sink for atmospheric C through the carbonation of peridotite (i.e., ophicarbonate formation) during serpentinization^11–13^ or the precipitation of hydrocarbons accompanying, for example, Fischer–Tropsch Type reactions^14^. In addition, during subduction, these rocks have the potential to regulate redox-sensitive elements, such as carbon^15^. As such, the high fO_2_-H_2_O rich fluids released during serpentinite dehydration promote the oxidation and leaching of carbon in percolated lithologies during fluid transfer e.g., refs. ^7,15–17^. Although several studies have stressed the role of external serpentinite-derived fluids for the remobilization of carbonate in percolated lithologies (structurally overlying slab crust) e.g., refs. ^18,19^, the fate of both inorganic and organic carbon stored in serpentinite and the carbon isotope signature of serpentinite-derived fluids at eclogitic conditions remains difficult to address^16,20^.

**Fig. 1:**
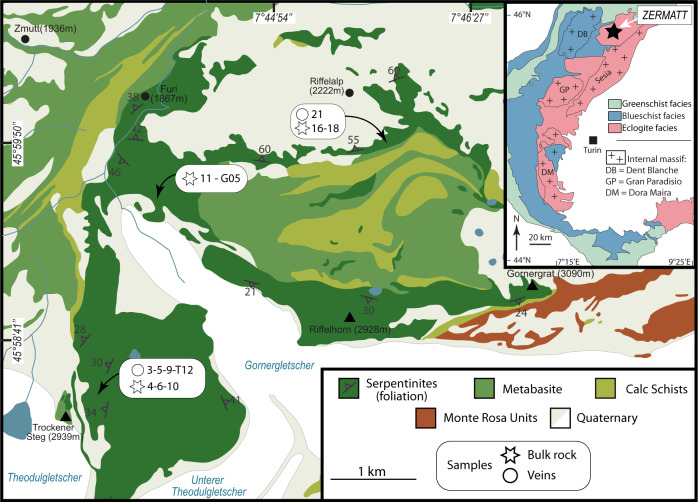
Simplified geological map and sample localization in the investigated area of the Zermatt-Saas meta-ophiolite. Upper inset shows the Zermatt body in the frame of the alpine metamorphic facies.

The Zermatt-Saas meta-ophiolite represents a ~30 km thick section of Jurassic Liguro-Piemontese oceanic lithosphere (Fig. 1), which underwent subduction to ~75 km depth during the Eocene and subsequent exhumation as part of the Alpine orogeny^21^. The massif comprises a full sequence of ophiolitic lithologies, including serpentinized ultramafic rocks, metabasites, and associated metamorphosed marine sediments (Fig. 1)^22,23^. This meta-ophiolite records a complex petrological evolution, from crustal formation and hydration in a mid-oceanic ridge environment to devolatilization at eclogite facies conditions, and finally exhumation from amphibolite to greenschist facies conditions during alpine collision^24^. We focused on serpentinite lithologies exposed in Switzerland, in the area adjacent to the town of Zermatt (Fig. 1). These rocks record UHP dehydration that occurred during alpine subduction, illustrated by the existence of Ti-chondrodite (TiCho) and/or Ti-clinohumite (TiChu) associated with metamorphic olivine (Ol) and antigorite (Atg)^25–27^. Although there are some discrepancies regarding the conditions of serpentinite dehydration in the massif, these are estimated to lie between 2.2 and 2.8 GPa, and between 550 and 650 °C. These conditions are consistent with those recorded by the Zermatt-Saas metagabbros ranging from 1.9–3 GPa and 530–650 °C, e.g., refs. ^23,28–30^ and in agreement with the discovery of microdiamonds and coesite in Zermatt-Saas metasedimentary rocks (Cignana Lake^25,31^). The whole massif experienced retrograde reactions and associated deformation, often obscuring the mineralogy relating to peak metamorphic conditions, with the generation of tremolites and serpentines overprinting the UHP minerals.

Here we present in situ and bulk-rock isotopic (O, C, and Sr) data on carbonate-bearing eclogitic veins and meta-serpentinites found in the Zermatt-Saas meta-ophiolite. We show that these veins are derived from fluids released during serpentinite dehydration at eclogite facies and that the presence of fluid-derived carbonates demonstrates the loss of isotopically heavy carbonate from the host serpentinite. Whereas the elemental and isotopic composition of organic carbon remains as graphite-like carbonaceous compounds in the residue and is relatively unchanged. These observations constitute natural evidence of serpentinite-hosted carbonate devolatilization accompanying slab mantle dehydration, suggesting that deep recycling of the serpentinized slab mantle leads to the preferential burial of organic carbon relative to carbonate, which may, at least in part, contribute to the light carbon isotopic signature of ocean island basalts e.g., ref. ^32^ and the isotopic diversity of diamond C e.g., refs. ^10,33^.

## Results

### High-pressure veins

**Fig. 2:**
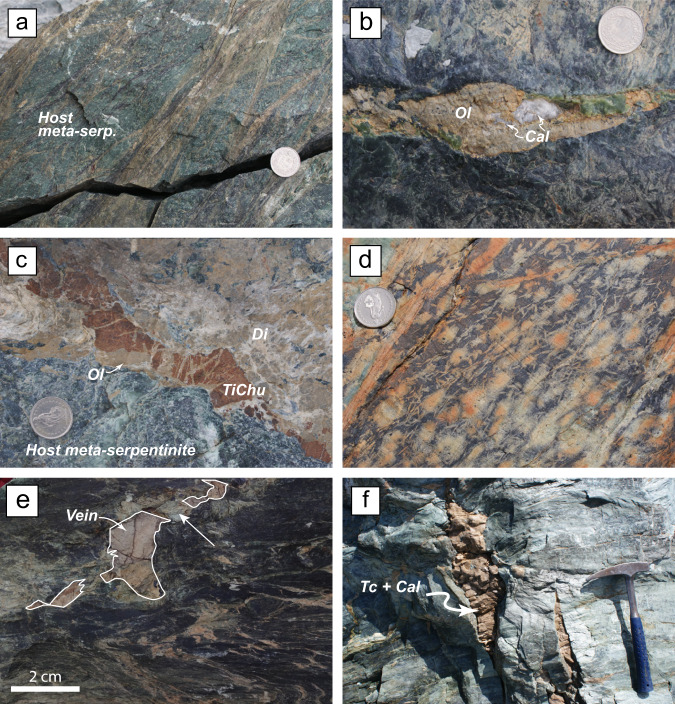
Field pictures of Zermatt-Saas meta-serpentinites and veins. **a** Meta-serpentinite showing a dense and anastomosed network of brown metamorphic veins, made of olivine (Ol), diopside (Di), chlorite (Chl), and magnetite (Mag) (sample ZE17-18: host meta-serpentinites) contemporaneous of the main deformation D2. **b** Sample Trock16-12 showing a pluri centimetric vein filled with cm size calcite grain in an olivine (brown) matrix. Late lizardite overgrowing the vein (light green patch below the coin) **c** Brecciated veins made of Ti-Clinohumite (TiChu), Ol, and calcite with fibrous Di; forming millimeter to centimeter blade. **d** Sample 11, showing coalescing lath (dendrite) and haloes (spherolite) of olivine (retromorphosed to Serp.) and chlorites in a black matrix made of Atg. **e** Ol-TiChu-Cal vein dissected and boudinaged by a late D3 deformation. White arrow points to fibrous chrysotile growing in the boudin’s neck. **f** Tension gashes made of talc (Tc) and calcite (Sample ZE17-09) crosscutting the main serpentinite fabric.

The Zermatt serpentinites were variably deformed, ranging from massive to foliated, during a complex polyphase metamorphic history^34^. Relicts indicative of oceanic metamorphism and deformation are rare in the serpentinites and most often overprinted by high-pressure metamorphic events. The oceanic stage is marked by the occurrence of relict mantle assemblages (olivine, pyroxenes, and spinels) overprinted by low-temperature fluid mediated metamorphism, typified by the occurrence of lizardite^35^, the low-temperature form of serpentine. The subduction-related metamorphic history is marked by the development of antigorite, olivine, and TiCho/TiChu. These features are overprinted by the main mesostructures of the massif acquired near the subduction-related metamorphic peak D2^34^, which is overall expressed as a strong foliation and by the crystallization of orientated Atg–Ol–TiCho-TiChu-Mgt ± diopside (Di) and chlorite (Chl) in foliated serpentinites (Fig. 2a, b). This deformation is often marked by C/S planes and associated Riedel shear structures that drove fluid percolation and crystallization leading to D2 related vein formation (Fig. 2a–c). These fluids are most likely derived from serpentinite devolatilization, for which there is evidence in massive outcrops (Fig. 2d). All the previous structures can be reworked by exhumation-related deformation and recrystallization, with the appearance of late folds and crenulation (Fig. 2e), associated with amphibole and low-temperature serpentine crystallization (e.g., chrysotile and/or lizardite)^34,36^. In some places, veins and tension gashes, with talc and calcite fillings crosscut all the previous features (Fig. 2f) and are interpreted to form during late-stage exhumation^27^.

**Fig. 3:**
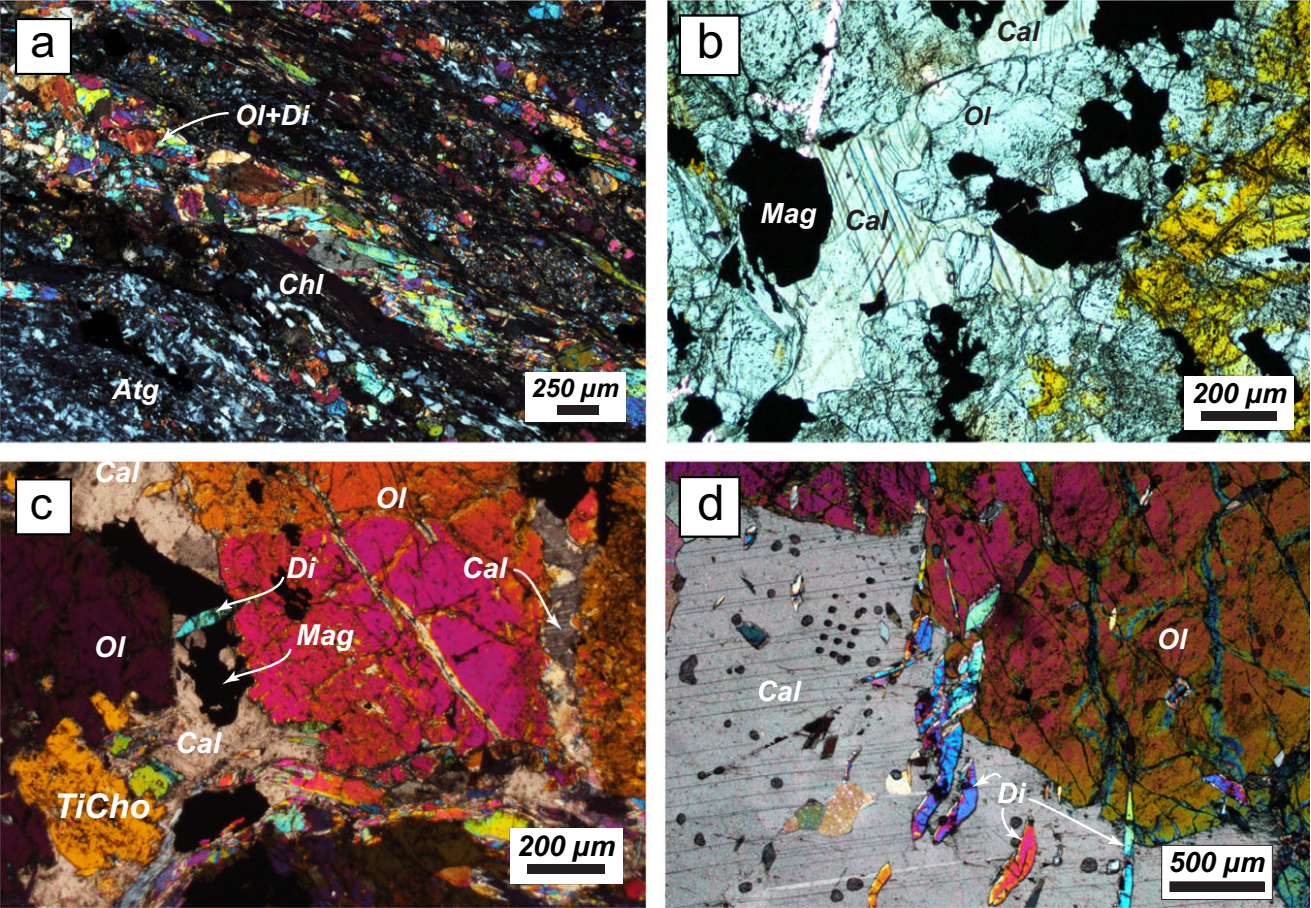
Vein thin sections pictures. **a** Plane polarized view of the veins observed in Fig. 2a showing a Ol ± Di ± Chl vein crosscutting the Atg bearing serpentinite. **b** Interstitial calcite, olivine, and magnetite in sample ZE17-21, also showing olivine replacing Ti-clinohumite (orange mineral on the right side). **c** Sample ZE17-05, with interstitial calcite, magnetite, and diopside that overprint olivine replacing TiCho. **d** Sample Trock16-12 showing the relationships between olivine and calcite, and the overprint by minute diopside.

The veins related to the D2 event are widespread throughout the ultramafic rocks and are dominated by a paragenesis of Ol, TiChu, TiCho, Chl ± Mgt, and/or ilmenite (Ilm), symptomatic of HP (e.g., Br + Atg → Ol + H_2_O) to UHP (e.g., TiChu + H_2_O → TiCho + Atg + Ol) reactions, associated with calcite and diopside (Fig. 2b, c). The presence of olivine replacing TiCho and TiChu (Fig. 3b, c), as well as the structural habit of the veins, which are often present in shear bands related to the main episode of deformation of the massif (D2 event, Fig. 2a), places the *P-T* formation conditions of the veins at eclogite facies, during serpentinite dehydration^26,27,34^. Texturally, calcite is found crystallizing at grain boundaries, replacing olivine (Fig. 3c), or displays a facetted contact with olivine (Fig. 3d) showing that both calcite and olivine ultimately precipitated from the same fluid at high pressure. Diopside crystallizes as a sub-automorphic millimeter to centimeter blades overprinting all minerals (Fig. 3c, d). When diopside is abundant, the veins display brecciated textures with a diopside matrix dismembering olivine and TiChu clasts (Fig. 2c). Such observations suggest that diopside crystallized at the expense of the calcite-olivine-TiChu-TiCho paragenesis. We interpret the calcite to be fluid derived and to have formed together with the symptomatic eclogitic minerals during serpentinite dehydration. We did not find any evidence for aragonite, but as the polymorphic transition is highly reversible, aragonite could have readily transformed to calcite during the P-T path followed by the massif^37,38^. Indeed, the absence of aragonite is not uncommon in HP or UHP terrains and can be attributed either to back reacted crystals during massif exhumation, or metastable HP calcite polymorphs that may dominate Ca-carbonate forms in HP to UHP conditions^39^. Indeed, Raman spectroscopy (see Supplementary Fig. 1) shows that most of the crystals are calcite *s.s*., but one spectrum could be interpreted as a CaCO3 III HP-polymorph. Based on the peak shifts, we can estimate the pressure of crystallization of this calcite polymorph to be close to 2–2.5 GPa^40^, in accordance with vein formation under eclogitic conditions during serpentinite devolatilization.

Near the contact with the overlying crustal lithologies, calcite is also found associated with talc in pluri-centimeter tension gashes crosscutting the serpentinite foliation (Fig. 2f), as well as in pods in the serpentinites (thereafter referred to as talc-calcite pods). Those are most likely related to fluid circulation during the massif exhumation^27^.

### Meta-serpentinite hosts

**Fig. 4:**
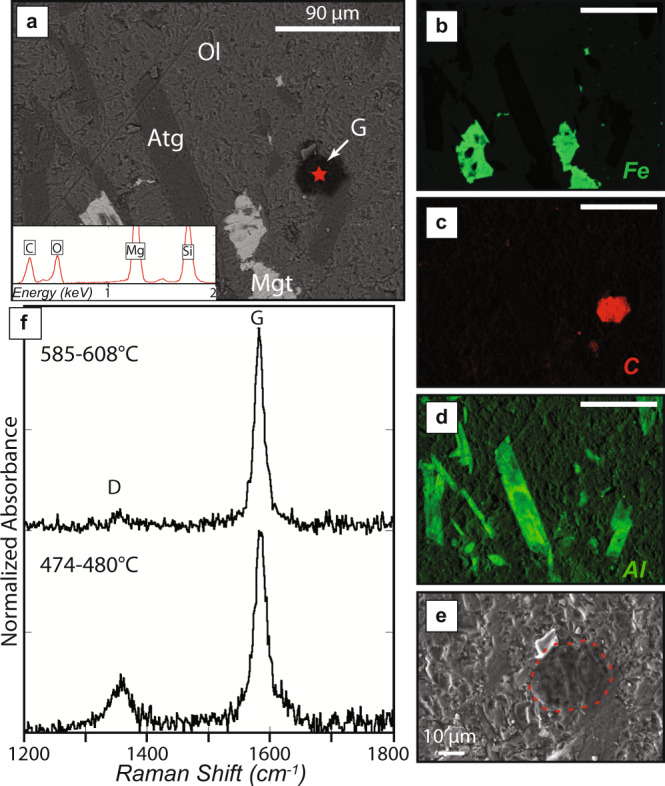
Carbonaceous matter in inclusions in a metamorphic olivine crystal (sample ZE17-16). **a**–**d** Back-scattered SEM image with associated EDS element maps (Fe, C, and Al) of metamorphic olivine containing inclusions of antigorite, magnetite, and graphite-like (G) carbonaceous matter. The red star corresponds to the EDS analysis of the carbonaceous matter, shown in a box on Fig. 4a. **e** SEM in lens images of graphite-like (G) carbonaceous matter showing that it is within the olivine crystal. **f** Raman spectra of graphite-like carbonaceous matter.

The meta-serpentinite hosts are carbonate-free (i.e., no carbonate is observed in the thin section). They are mainly made of Atg-Ol-Mgt assemblages associated with minor TiChu-TiCho^27^. The meta-serpentinite forming olivine contains numerous mineral inclusions (Fig. 4) consistent with a metamorphic origin. Based on high X_Mg_ of olivine, Kempf et al.^27^ suggested that these crystallized at temperatures nearby 500–550 °C, under eclogitic conditions. Careful SEM and Raman spectroscopy mapping of metamorphic olivine grains reveals the existence of endogenous carbonaceous compounds in inclusion within olivine (Fig. 4b–e). Their Raman spectra are characterized by a first-order region comprising a sharp and intense band nearby 1585 cm^−1^ and a broad and small band nearby 1355 cm^−1^ corresponding to graphite (G) and disordered (D) carbon, respectively (Fig. 4f)^41^. These spectra are similar to those reported in graphite-like carbonaceous compounds from eclogitic meta-sediments^41^ where the carbonaceous material is derived from biological organic matter originally present in sedimentary rock. Indeed, by using conventional Raman thermometry^41,42^, temperature estimates range from 470 to 610 °C. These values are close to the peak temperatures of the massif suggesting that these carbonaceous compounds are likely to be derived from biological organic matter originally present in the serpentinites prior to subduction. However, it must be noted that the scattering of temperature estimates in a single sample (Fig. 4f) is larger than usually observed in metasedimentary rocks (50 °C maximum^43^). This uncertainty may reflect the existence of heterogeneous carbonaceous compounds in serpentinites prior to subduction. Indeed, these rocks can incorporate a large variety of not only biologic but also abiotic carbonaceous compounds, including hydrocarbons^44^, amino acids^45^, and condensed carbonaceous matter^46^. The thermal evolution of these carbonaceous compounds during subduction might differ from that observed in metasedimentary rocks. Similarly, recent thermodynamic studies^47^ have also suggested that these carbonaceous compounds can be stable during subduction and might therefore also be formed at high pressure during serpentinite dehydration reactions. Considering this, the disorder observed in the Raman spectra might either correspond to the formation of aromatic hydrocarbons with complex aliphatic chains (e.g., carboxylic groups) or to different thermal evolution of complex abiotic carbonaceous compounds (e.g., hydrocarbons, amino acids, and condensed carbonaceous matter) during subduction.

### Meta-serpentinite compositions

**Fig. 5:**
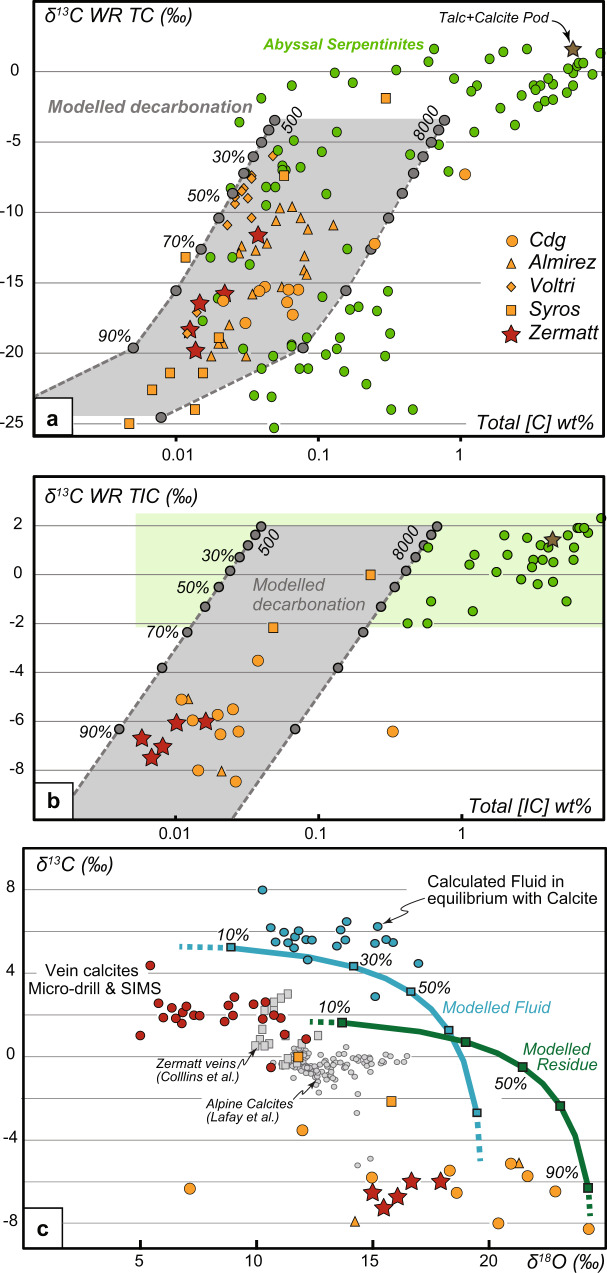
Carbon bulk-rock and calcite isotopic and elemental variations. **a** Bulk rock total carbon isotopic composition (δ^13^CTC) versus total carbon content for the Zermatt samples, compared to abyssal^12,57^ and subduction-related serpentinites and meta-serpentinites/meta-peridotites from the Almirez (Spain), Voltri (Italy), Syros (Greece), and Cima di Gagnone (Cdg, Switz.) blueschist and eclogite massifs. **b** Bulk rock inorganic carbon isotopic composition (δ^13^CTIC) versus inorganic carbon concentrations of the same sample suite. Green box = possible extent of abyssal serpentinite composition. **c** O and C isotopic composition (δ^18^OTIC and δ^13^CTIC) of the calcites present in veins (red dots) and the bulk meta-serpentinites (red stars). Bulk veins from Zermatt^55^, calcite compositions of Alpine samples^50^ for comparison. On top of the three figures, a simple Rayleigh decarbonation model at 600°C is presented with the % of devolatilization (see Method) and calculated fluid composition in equilibrium with measured calcites (blue dots).

**Fig. 6:**
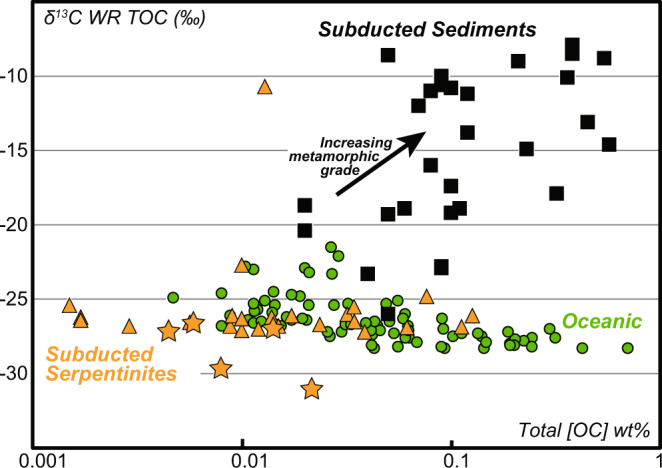
Bulk rock δ^13^CTOC versus [CTOC] composition. Comparison between subduction-related (meta-) serpentinites (Orange; Stars = Zermatt, this study), abyssal peridotites (Green) (data as in Fig. 3) and subducted eclogitic meta-sediments^64^.

Overall, the total carbon contents of the meta-serpentinites (127 to 379 µg/g) and their δ^13^C_TC_ (−19.82 to −11.65‰) (Table S1) are within the range of those observed in abyssal serpentinites (Fig. 5a). Nonetheless, as shown below, their C isotopic characteristics allow a clear distinction between eclogite meta-serpentinites and abyssal serpentinites. Although the inorganic compounds have a rather uniform δ^18^O_TIC_ (from +15.00 to +17.95‰), similar to other alpine serpentinites (+10; +18‰, Fig. 5), these values are lower than present-day abyssal serpentinites δ^18^O_TIC_ (~ +30‰; e.g., ref. ^12^). Furthermore, the Zermatt meta-serpentinites have low Total Inorganic Carbon (TIC) concentrations, ranging from 58 to 161 µg/g, and low δ^13^C_TIC_, between −7.48 to −6.01‰, compared to abyssal serpentinites (−2.0 and +2.3‰,^12^, Fig. 5). Conversely, the Total Organic Carbon (TOC) concentrations (44–218 µg/g) and δ^13^C_TOC_ (−31.11 to −26.65‰) of Zermatt meta-serpentinites are comparable to those of abyssal serpentinites (47–7000 µg/g; δ^13^C_TOC_ = −21.5, −28.3; Fig. 6). Altogether, the C concentrations and isotopic characteristics of the Zermatt meta-serpentinites are similar to those of UHP meta-serpentinites (meta-peridotites) from other eclogite ultramafic massifs (Fig. 5,^10,20^). One serpentinite devoid of metamorphic veins, has a ^87^Sr/^86^Sr of 0.707433 (±02, 2 s.e.), and the talc-calcite pods have similar Sr isotopic composition (^87^Sr/^86^Sr = of 0.706825 (08)), all within the range of other alpine meta-serpentinites (0.7039 < ^87^Sr/^86^Sr < 0.7105, 0.7060 in average^25,48,49^).

### Vein isotopic composition

In the veins, the in situ calcite analyses show a restricted range of δ^13^C, from −0.15 to +2.85‰ (except 2 points at +3.53 and +4.36‰ in TROCK16-12) and a wide range of δ^18^O, spanning between +5.0 and +12.1‰, which is lower than any other calcites found in alpine terranes e.g., ref. ^50^ (Fig. 5c and Table S1) or during sub-surface alteration of ultramafic terrains^51,52^. In TROCK16-12 and ZE17-05, olivine and diopside compositions are relatively constant, (+3.07 < δ^18^O_olivine_ < +5.74‰; +2.34 < δ^18^O_diopside_ < +4.67‰). In sample ZE17-21, which does not contain diopside, the olivine and magnetite show variable isotopic compositions (−0.80 < δ^18^O_olivine_ < +3.60‰; −4.59 < δ^18^O_magnetite_ < +7.19‰). These variations most likely reflect different states of mineral-mineral equilibrium, and or different states of equilibrium with an evolving fluid composition. The temperature estimates derived from O isotopic equilibrium between mineral species^53^ are consistent with the metamorphic conditions of the massif. In detail, Ol-Cal equilibrium temperature estimates range between 420 and 640 °C, with an average of 550 °C, in the same range as Ol-Mgt and Cal-Mgt (in ZE17-21: 460 and 520 °C respectively); whereas Ol-Di and Cal-Di equilibrium estimates show a restricted temperature range around 400 °C, which most likely reflects the late character of diopside, which is observed as overprinting olivine or calcite, and other phases crystallizing in the veins. Overall, the ^87^Sr/^86^Sr of vein calcite is homogeneous within error (weighted mean of all measurements: 0.7067(18)), with ZE17-21 having the highest value (0.7081(26)) and is similar to that of meta-serpentinites bulk rock (0.707433(02)) and other alpine meta-serpentinites^25,48,49^. The δ^18^O and δ^13^C of the calcite of the talc + calcite assemblage in the tension gashes are nearly identical to those of the talc + calcite pod, close to the maximum δ^18^O of the calcite veins. Furthermore, the pod has an ^87^Sr/^86^Sr value of 0.706825 (08) identical within error to the vein calcite. These talc bearing samples, a paragenesis typical of low-temperature fluid conditions^54^, notably present in brittle tension gashes that crosscut all structures, most likely represent low-temperature fluid-derived precipitates formed during the exhumation of the massif^27,55^.

## Discussion

Along average slab thermal gradients, carbonates are remarkably stable within the subducting oceanic lithosphere^56^, and only a substantial percolation of H_2_O-rich fluids can promote significant carbon remobilization in sediments, oceanic crust, and mantle lithologies through leaching^5–7^. It has recently been predicted, based on thermodynamic models, that the large volumes of fluids released during serpentinite dehydration can promote such mobilization of slab mantle carbon in fluids^16^. Here we document such a process, where serpentinite-hosted C has been remobilized in fluids during dehydration at eclogitic conditions under oxidizing form (e.g., CO_2_). We further show that serpentinite devolatilization fractionates the C compounds, where the inorganic C would be remobilized, leaving the organic C as a graphite-like carbonaceous compound (Fig. 4), to be recycled in the deep mantle.

Total C concentrations and isotope compositions of abyssal serpentinites are considered to reflect a two-component mixing, between an organic carbon endmember (δ^13^C −25‰) and an inorganic carbonate endmember (δ^13^C + 5‰),^57^. Although comparable in terms of concentration and isotopic values, high-pressure meta-serpentinites tend to display lower carbon concentration and δ^13^C_TC_ relative to abyssal serpentinites and their composition cannot be explained by a simple mixing between carbonate and organic carbon. Indeed, the C_TIC_ and δ^13^C_TIC_ in meta-serpentinite are very low relative to abyssal serpentinites (Fig. 5b). It must be noted that little is known about the δ^13^C_TIC_ in abyssal serpentinites. In particular, there is a lack of data regarding the δ^13^C_TIC_ in low [C_TIC_] abyssal serpentinite (<0.5 wt%; Fig. 3b), which hampers our understanding of the inorganic carbon cycle from mid-oceanic ridges to subduction zones processes. Although inorganic carbon commonly displays high δ^13^C_TIC_ values (>0‰), the serpentinization of abyssal peridotites at mid-oceanic ridges leads to complex redox processes (i.e., methane oxidation or carbonate reduction) that might influence the δ^13^C_TIC_ in unexpected ways. Indeed, several studies on sub-surface alteration of ultramafic terrains^51,52^ report the formation of carbonates with low δ^13^C_TIC_ in ultramafic host paleo-hydrothermal systems. These values are attributed to the percolation of hydrous fluids with a light isotopic signature, potentially derived from decarboxylation of organic matter-rich sediments, at low temperatures. Here, the low δ^18^O_TIC_ of calcite veins associated with meta-serpentinites are incompatible with such low-temperature hydrothermal processes, thus inferring that the δ^13^C_TIC_ of the meta-serpentinite do not reflect a hydrothermal origin and/or late processes accompanying the exhumation of the massif during alpine collision. This is further supported by the ^87^Sr/^86^Sr of both the veins and host meta-serpentinites that exclude a contribution of organic matter-rich sediments. Because sediments have ^87^Sr/^86^Sr > 0.712, and meta-serpentinites have very low Sr concentrations, any interaction between meta-serpentinites and sediment-derived fluids prior or during subduction would give rise to radiogenic Sr isotopic values, which are not observed here. Rather, the meta-serpentinite host rock Sr isotopic composition is inherited from the interaction of a depleted mantle (DMM) with Jurassic seawater during serpentinization of the protolith, giving rise to the observed ^87^Sr/^86^Sr values around ~0.7074, which is a similar value to other alpine meta-serpentinite that have not interacted with sediment-derived fluids (0.7046 < ^87^Sr/^86^Sr < 0.7083, 0.7060 in average^25,48,49^). Under these conditions, the δ^13^C_TIC_ of the protolith is expected to be formed in equilibrium with seawater and therefore to display a heavy signature (δ^13^C_TIC_ > 0‰). Hence, the low [C_TIC_] and δ^13^C_TIC_ in UHP meta-serpentinites can be interpreted as evidence for the leaching of isotopically heavy C in metamorphic fluids during meta-serpentinite dehydration. As such, the carbonate-bearing veins are a good candidate to represent the crystallization products of such fluids. Indeed, although the carbonates are found in veins associated with an eclogitic paragenesis comprising olivine-TiChu-TiCho-magnetite-chlorite (Figs. 2, 3), they, nevertheless, have Sr isotopic values similar to the meta-serpentinite host rock. As the vein carbonate, isotopic values represent the composition of the fluid from which they crystallized, and they are identical within error to the host meta-serpentinites, we thus interpret the fluid to mainly arise from the dehydration of adjacent meta-serpentinite. Such an interpretation is further strengthened by the B isotopic composition of antigorite and secondary olivine of Zermatt meta-serpentinites that is also incompatible with the input of external fluids^58^. Overall, these observations suggest that the carbonate-bearing veins crystallized from a fluid derived from the devolatilization of the meta-serpentinite. Accordingly, the eclogitic vein-hosted carbonate displays a complementary high δ^13^C value relative to that of the meta-serpentinite host rocks.

To establish the complementarity between the isotopic signatures of the vein calcite and the meta-serpentinite host rock, we use a simple Rayleigh devolatilization model (Fig. 5), whereby the serpentinite inorganic carbon stored as calcite is remobilized during serpentinite dehydration. In this model, the elemental and isotopic behavior of carbon is approximated to an equilibrium between carbonate and CO_2(aq)_ (See Appendix B for details). This assumption allows calculation of the fluid C and O isotopic composition to be compared to the fluid in equilibrium with the vein carbonate. Ocean floor serpentinites are shown to contain a wide range of [C_TIC_], between 50 and 96150 µg/g and as such we show in Fig. 4 two models which differ in starting [C_TIC_], at 500 and 8000 ppm, which encompasses most of the concentrations measured in abyssal peridotites (green box of Fig. 5b). The chosen starting values of +2 for δ^13^C_TIC_ and +20 for δ^18^O, are representative of the average Ligurian Ocean calcites values found in unmetamorphosed alpine carbonated serpentinites (Fig. 5), whereas the chosen value of −25‰ for δ^13^C_TOC_ is the average value of abyssal serpentinites that ranges between −28.3 to −21.5‰. Note that changing the starting δ^13^C_TOC_ has little impact on the model results, as for the TIC/TOC starting ratio. The model shows that the δ^13^C_TIC_ and [C_TIC_] of the meta-serpentinites (Fig. 5b) can be explained by a nearly complete loss of inorganic carbon (>90%) as a result of devolatilization up to 600 °C (the modeled temperature). The calculated fluid composition in equilibrium with the vein calcite at those conditions ranges between +3.1 to +7.9‰ for δ^13^C and between +10.2 to +16.9‰ for δ^18^O (Fig. 5c), which encompasses the trend of the modeled fluid (Fig. 5c) up to 50% devolatilisation (Fig. 5c). This typifies snapshots of the devolatilisation process, whereby the measured calcites represent early fluids, saturated in carbonate, whereas later fluids with a much higher H_2_O proportion will not be recorded through carbonate precipitation. In Fig. 5c, the modeled residue has a higher δ^18^O (~8‰) at 90% devolatilization than the measured meta-serpentinite carbonate compound, most likely reflecting the model assumption that do not take into account the O isotopic fractionation between the H_2_O fluid species and the carbonate but only the CO_2_ fluid species (see methods). The devolatilization trend is thus typified by the modeled fluid composition released during devolatilization confirming that the UHP-vein calcite could be derived from a C-bearing fluid released from serpentinites at high temperature and pressure.

Taken together, this modeling suggests that the serpentinite inorganic C has been almost completely remobilized in the fluid during dehydration. As for most subduction, the slab thermal architecture implies serpentinite devolatilization at a depth that would imply that this fluid was able to participate in mantle wedge melting^59,60^, and as such the involvement of C-bearing fluids with a heavy δ^13^C potentially may account for the positive deviation of arc magmas to higher δ^13^C than the MORB mantle e.g., refs. ^61,62^.

Our data shows a low TIC and δ^13^C_TIC_ in the meta-serpentinites, which we interpret as being the results of C loss during fluid mediated devolatilization, where the calcite bearing veins represent the crystallization product of this fluid. As such subducting serpentinites (i.e with up to ~1 wt% [TC], Fig. 5) may have lost as much as 90% of their original C, but the striking feature of this process is the behavior of the inorganic C. A simple isotopic exchange between organic C and inorganic C could potentially explain a low δ^13^C_TIC_ e.g., ref. ^12^, but this would imply a concomitant increase in δ^13^C_TOC,_ which is not observed here, making this solution unlikely. Indeed, Fig. 6 shows that HP and UHP meta-serpentinites have a near-constant δ^13^C_org_ for a wide range of C_org_. Given that most of the samples display an abyssal like C_org_ or δ^13^C_org_, it is reasonable to propose that most of the serpentinite organic carbon with highly negative δ^13^C values preserved as graphite-like carbonaceous compounds (Fig. 4) is transferred towards the deep mantle.

Recent stable isotope studies of oceanic island basalts (OIB) have shown that the genesis of this magmatism involves the melting of a C-bearing component recycled in the convecting mantle by subduction^32,63^. The presence of such a C component is further emphasized by diamond isotopic composition e.g., ref. ^33^. Based on the heavy δ^13^C_TC_ and δ^66^Zn of OIBs, these studies also propose that carbon must be mostly present as the organic form in the source of these magmas. This requires a preferential release of inorganic C at the volcanic arc and deep recycling of organic C during subduction. We show here that the devolatilization of serpentinites during subduction can control such decoupling behavior between organic and inorganic carbon during subduction and therefore exerts a major control on global C cycling.

Although the inorganic C distribution in the oceanic lithosphere is thought to be mainly controlled by sediments, the distribution of organic C is much less well constrained. The δ^13^C_TOC_ of metasedimentary rocks tends to increase during subduction, ranging from −23.3 and −8.5‰ in eclogitic metasedimentary rocks^64^. These authors interpret such an increase through the isotopic re-equilibration between organic and inorganic carbon with increasing metamorphic grades, and further show that carbonate-rich samples have the largest increase in δ^13^C_TOC_, while those that have low carbonate concentration tends to have δ^13^C_TOC_ typical of oceanic organic matter (Fig. 6). Both eclogitic meta-ultramafic and meta-sediments, therefore, display comparable ranges of [C_TOC_] (Fig. 6). However, for a given [C_TOC_] meta-serpentinites display low δ^13^C_TOC_ suggesting that the recycling of serpentinites is a more efficient means to generate heterogeneities with low δ^13^C_TOC_ in the deep mantle. Given the large volume of serpentinized mantle recycled into the deep mantle by subduction, it is reasonable to propose that recycling of serpentinite could be a major source of the organic carbon heterogeneities observed in the deep mantle and thus the organic carbon detected in the geochemical signature of OIB.

## Methods

### Analysed material

We selected 4 UHP veins containing olivine, calcite ± diopside, TiChu, TiCho, magnetite, and ilmenite for both in situ and bulk oxygen (δ^18^O), C (δ^13^C), and Sr (^87^Sr/^86^Sr) isotope analyses. Secondary Ion Mass Spectrometry (SIMS) analyses were performed on calcite for δ^18^O, δ^13^C, and ^87^Sr/^86^Sr, and on olivine, diopside and magnetite, when present, for δ^18^O. For one vein sample (ZE17-03) containing centimeter-sized calcite, micro-drilled bulk analyses by conventional mass spectrometry were also performed for δ^18^O and δ^13^C, as well as the calcite associated with talc hosted within tension gashes (ZE17-09).

To complete our dataset, five meta-serpentinite host rocks, as well as one talc and calcite pod (ZE17-10), were analyzed for bulk δ^18^O, δ^13^C, and two meta-serpentinites were analysed for ^87^Sr/^86^Sr isotope ratios. Carbon and oxygen isotope analyses of bulk rock meta-serpentinites were performed for both inorganic (TIC: Total Inorganic Carbon) and organic (TOC: Total Organic Carbon) carbon compounds in addition to total carbon (TC).

### Carbon characterization

Sample preparation for carbon characterization was performed at the Institut de Physique du Globe de Paris (IPGP, France). Rock samples were sawn with a Cu-blade and sterile ultrapure water to extract the inner core, free of possible post-sampling contamination. The inner core was then manipulated using clean pliers, thinned and polished on both faces (down to a thickness of tens of micrometers) with pure ethanol using alumina polishing disks without any use of resin or glue.

Raman data were obtained at IPGP, on resin-free samples with a Renishaw InVia spectrometer using the 514 nm wavelength of a 20 mW argon laser-focused through an Olympus BX61 microscope with an x100 objective (numerical aperture: 0.9, respectively). This configuration yields a planar resolution close to 1 µm. The laser power delivered at the sample surface was 0.5 mW with integration times of 100 s, well below the critical dose of radiation that can damage the carbonaceous matter (Ménez et al., 2012). Spectra were fitted using peakfit© software based on the method of Beyssac et al. (2002), with a T uncertainty of about 50 °C

SEM observations were performed at the IPGP using a Zeiss Auriga FEG-FIB field emission scanning electron microscope. Samples were Au-coated. Images were collected using a backscattered electron detector (BSE) and secondary electron (SE) detector at high and low currents, respectively, with accelerating voltage ranging from 10 to 15 kV. Energy-dispersive X-ray spectrometry (EDXS) measurements were performed at 15 kV accelerating voltage using a Bruker detector (Nano GmbH, Germany).

### Bulk rock and bulk calcite analyses

Carbon and oxygen isotopic compositions of calcite were determined by using an autosampler Gasbench coupled to a Thermo Scientific MAT 253 isotope ratio mass spectrometer (IRMS) at the CRPG UMR 7358 CNRS-UL, Vandoeuvre les Nancy. For each sample, an aliquot between 1 to 100 mg of powder was reacted with 2 mL of supersaturated orthophosphoric acid at 70 °C for at least 10 h under a He atmosphere. Carbon and oxygen isotopic compositions of the produced CO_2_ were then measured with a Thermo Scientific MAT 253 continuous flow isotope ratio mass spectrometer. Values are quoted in the delta notation in ‰ relative to V-PDB for carbon and converted to V-SMOW for oxygen. All sample measurements were adjusted to the internal reference calibrated on the international standards IAEA CO-1, IAEA CO-8, and NBS 19. The reproducibility was better than 0.2‰. Carbonate contents of the samples were determined by comparison with four internal standards consisting in fine-grained marine sediments from the Bay of Bengal and routinely included during the analysis: (i) BR 516: CaCO_3_ = 3.49 wt%, (ii) BR 8107: CaCO_3_ = 6.21 wt%, (iii) CA 10-8: CaCO_3_ = 11.94 wt% and (iv) NAG 7-RT: CaCO_3_ = 17.22 wt%. Errors (2σ) on carbonate content are estimated to be 5 and 30% for samples containing more and less than 0.1 wt% CaCO_3_, respectively. TIC was then calculated following TIC (%) = wt% CaCO_3_/100 * 12.

Determination of the total carbon concentrations (TC) and isotopic composition (δ^13^C) of the samples were performed online using the Thermo Scientific EA IsoLink IRMS System at CRPG laboratory (Nancy, France). Samples were wrapped in tin capsules (~30 mg) and then combusted at 1020 °C in a combustion reactor consisting of quartz tube filled with chromium oxide, pure copper, and silvered cobalt oxide. Produced gases (N_2_ and CO_2_) were separated on a chromatographic column maintained at 70 °C and carbon isotopic composition of the produced CO_2_ was then measured with a Thermo Scientific Delta V Advantage continuous flow isotope ratio mass spectrometer. Carbon isotopic compositions were determined by comparison with two internal and two international standards routinely included during the analysis: (i) BFSd (δ^13^C = −21.5‰), (ii) CRPG_M2 (δ^13^C = −24.98‰), (iii) NBS22 (δ^13^C = −30.03‰), and (iv) USGS24 (δ^13^C = −16.1‰). Values are quoted in the delta notation in ‰ relative to V-PDB and the reproducibility was better than 0.2‰. Errors (2σ) are expected to be lower than 0.5‰ for δ^13^C. Two internal and two international standards were used to calculate the total carbon concentration of the samples (TC): (i) BFSd (0.53 wt% C), (ii) CRPG_M2 (0.408 wt% C), (iii) GSJ JG-3 (0.012 wt% C), and (iv) USGS PCC-1 (0.042 wt% C). Errors (2σ) on [C] are estimated to be lower than 10%.

Determination of the total organic carbon isotopic composition (δ13C) of the samples was performed online using the same procedure than for total carbon. Samples were previously decarbonated with HCl fumigation for at least 5 days at 65 °C. The fact that the samples have been previously decarbonated by HCl fumigation leads to large uncertainties for TOC (up to 50%). This is why we chose to calculate TOC by difference where TOC = TC – TIC.

For Sr analyses, 100 mg of fine-grained powder were digested into Teflon beakers using a mixture of concentrated ultrapure acid (HNO_3_ + HF). The beakers were placed on a hotplate for 2 days to dissolve the components of the sample. The complete digestion is done by using ultrapure HCl for 1 day. For Sr isolation, an extraction chromatographic separation technique by Sr-spec resin was used (Pin et al., 1997). The Sr isotopic compositions are measured on a thermoionisation spectrometer (Triton, Thermo Scientific). The instrumental mass bias was corrected by an internal normalization using ^86^Sr/^88^Sr = 0.1194. The NBS987 was used as a reference standard with the reference value ^87^Sr/^86^Sr = 0.710259 ± 0.000019.

### In situ SIMS analyses

The Sr analyses were carried out at CRPG (Nancy, France) with the Cameca IMS 1280 HR. The primary O^−^ beam was set in Gaussian mode at an intensity of 50 nA and the spot size was ~25 μm. The secondary positive ions were measured in rectangular mode with a mass resolution (M/∆M) of 22000 to separate peaks of interest from isobaric interferences. The field aperture was set at 2500 µm, the transfer magnification at 80 µm, and the energy window at 30 eV. Masses of interest were measured by peak-jumping for 20 cycles on the axial electron multiplier as follows: 83.7 (3 s), ^84^Sr (3 s), ^40^Ca^44^Ca (3 s), ^85^Rb (8 s), ^86^Sr (16 s), ^87^Sr (16 s), and ^88^Sr (8 s). A pre-sputtering of 90 s and automatic centerings of mass, energy, and secondary beam were applied. Our in-house calcite standard CCcigA ([Sr] = 1061.9 ppm and ^87^Sr/^86^Sr = 0.708484 ± 0.000006) was used for the calibration of the instrumental mass fractionation. The typical analytical uncertainty on the ^87^Sr/^86^Sr ratio is ~0.3% relative (1σ) and the external reproducibility on the standard over the session is ~0.07% relative (1σ).

The C analyses were carried out at CRPG (Nancy, France) with the Cameca IMS 1270 E7. ^12^C^−^ and ^13^C^−^ ions produced by a Cs^+^ primary ion beam (~20 µm, ~4 nA) were measured in multi-collection mode using one off-axis Faraday cups for ^12^C^−^ and the axial electron multiplier for ^13^C^−^. In order to remove the ^12^CH^−^ interference on the ^13^C^−^ peak and to get maximum flatness on the top of the ^12^C^−^ peak, entrance and exit slits were adjusted to get an MRP of ≈5000 for ^13^C^−^ on the central EM. The multi-collection FC was set on slit 1 (MRP = 2500). The field aperture was set at 2000 µm, the transfer magnification at 107 µm, and the energy window at 40 eV. Automatic centerings of mass and secondary beam were applied. The total measurement time was 290 s (200 s measurement + 90 s pre-sputtering). We used our in-house calcite standard CCcigA (δ^13^C_PDB_ = 1.04‰) to correct the instrumental mass fractionation due to the matrix effect in samples. Typical count rates obtained on the calcite standard were 1.8 × 10^7^ cps for ^12^C and 2 × 10^5^ cps for ^13^C. The typical internal error was ≈0.3‰ (2σ) and the external reproducibility on the calcite standard CCcigA was ≈0.2‰ (2σ).

We measured the oxygen isotopic compositions with a CAMECA IMS 1270 E7 at CRPG-CNRS (Nancy, France). ^16^O^−^, and ^18^O^−^ ions produced by a Cs^+^ primary ion beam (~20 mm, ~4 nA) were measured in multi-collection mode using two off-axis Faraday cups (FCs) for ^16^O^−^ and ^18^O^−^. The multi-collection FCs were set on slit 1 (MRP = 2500). The field aperture was set at 2000 µm, the transfer magnification at 107 µm, and the energy window at 40 eV. Automatic centerings of mass and secondary beam were applied. The total measurement time was 210 s (150 s measurement + 60 s pre-sputtering). We used four in-house terrestrial standard materials (San Carlos olivine, CCcigA calcite, Charroy magnetite, and JV1 diopside) to correct the instrumental mass fractionation (IMF) due to the matrix effect in samples. Typical count rates obtained on the San Carlos olivine standard were 2 × 10^9^ cps for ^16^O, and 5 × 10^6^ cps for ^18^O. The typical internal error was ≈0.15‰ (2σ) for all of the standards and the external reproducibility was ≈0.2–0.5‰ (2σ) depending on the standard.

### Geochemical model

On top of the three panels of Fig. 5, a simple Rayleigh decarbonation model at 600 °C is presented, where carbonate-bearing serpentinite with an initial δ^18^O = +20‰; δ^13^C_TIC_ = +2‰; δ^13^C_TOC_ = −25‰; (80% of the total C is considered as inorganic) lose its carbonate component simultaneously with water (e.g., 10% devolatilization corresponds to F = 0.1 and residual carbonate = 90%). In A and B, the residual rocks modeled is presented with two models: one with a starting [C_TC_] of 500 ppm, the other with a starting [C_TC_] of 8000 ppm; the green box in B represents the range of oceanic [IC]. Fractionation factors calculated following^65,66^ (See Supplementary data for other details).

## Supplementary information

Supplementary Information

Description of Additional Supplementary Files

Supplementary Data 1

## Data Availability

All the data is available in the supplementary dataset.
