# Supplementary material for: Decoupling of inorganic and organic carbon during slab mantle devolatilisation
Source: Nat Commun. 2022 Jan 14;13:308. doi: 10.1038/s41467-022-27970-0 (PMC8760304; doi:10.1038/s41467-022-27970-0)
Supplement: Supplementary file 1 — Supplementary Information [file 41467_2022_27970_MOESM1_ESM.pdf]

Supplementary Figure 1

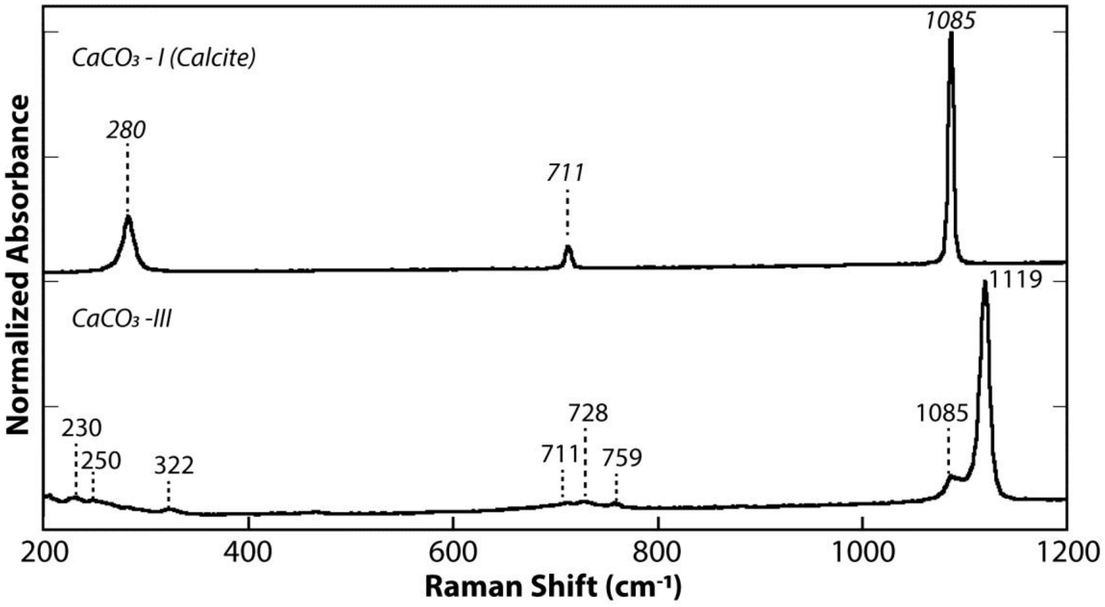

Raman spectra of two calcite grains in sample ZE17-03, one showing a spectrum that can be interpreted as a HP Polymorph
