# Supplementary material for: Decoupling of inorganic and organic carbon during slab mantle devolatilisation
Source: Nat Commun. 2022 Jan 14;13:308. doi: 10.1038/s41467-022-27970-0 (PMC8760304; doi:10.1038/s41467-022-27970-0)
Supplement: Supplementary file 2 — Description of Additional Supplementary Files [file 41467_2022_27970_MOESM2_ESM.docx]

Description of Additional Supplementary Files

Title: Supplementary Data 1

Description: All data and model
